# Recruitment of pre-dementia participants: main enrollment barriers in a longitudinal amyloid-PET study

**DOI:** 10.1186/s13195-023-01332-4

**Published:** 2023-11-02

**Authors:** Ilse Bader, Ilona Bader, Isadora Lopes Alves, David Vállez García, Bruno Vellas, Bruno Dubois, Mercè Boada, Marta Marquié, Daniele Altomare, Philip Scheltens, Rik Vandenberghe, Bernard Hanseeuw, Michael Schöll, Giovanni B. Frisoni, Frank Jessen, Agneta Nordberg, Miia Kivipelto, Craig W. Ritchie, Oriol Grau-Rivera, José Luis Molinuevo, Lisa Ford, Andrew Stephens, Rossella Gismondi, Juan Domingo Gispert, Gill Farrar, Frederik Barkhof, Pieter Jelle Visser, Lyduine E. Collij, on behalf of the AMYPAD consortium

**Affiliations:** 1 Alzheimer Center Amsterdam, Neurology, Vrije Universiteit Amsterdam, De Boelelaan 1118, 1081 HZ Amsterdam, The Netherlands; 2 Amsterdam Neuroscience, Neurodegeneration, 1081 HV Amsterdam, The Netherlands; 3 Radiology & Nuclear Medicine, Vrije Universiteit Amsterdam, Amsterdam UMC Location VUmc, 1081 HZ Amsterdam, The Netherlands; 4 Amsterdam Neuroscience, Brain Imaging, Amsterdam, 1081 HV The Netherlands; 5 Brain Research Center, 1081 GN Amsterdam, The Netherlands; 6 Gérontopole of Toulouse, University Hospital of Toulouse (CHU-Toulouse), 31300 Toulouse, France; 7 UMR INSERM 1027, University of Toulouse III, 31062 Toulouse, France; 8 Institute of Memory and Alzheimer’s Disease (IM2A) and Brain Institute, Salpetriere Hospital, Sorbonne University, 75013 Paris, France; 9 Ace Alzheimer Center Barcelona, Universitat Internacional de Catalunya (UIC), 08028 Barcelona, Spain; 10 Networking Research Center On Neurodegenerative Diseases (CIBERNED), Instituto de Salud Carlos III, 28029 Madrid, Spain; 11 Neurology Unit, Department of Clinical and Experimental Sciences, University of Brescia, 25123 Brescia, Italy; 12 Laboratory for Cognitive Neurology, Leuven Brain Institute, KU Leuven, 3001 Louvain, Belgium; 13 Institute of Neuroscience, Université Catholique de Louvain, 1200 Brussels, Belgium; 14 Department of Neurology, Clinique Universitaires Saint-Luc, 1200 Brussels, Belgium; 15 Gordon Center for Medical Imaging, Department of Radiology, Massachusetts General Hospital, Boston, MA 02155 USA; 16 WELBIO Department, WEL Research Institute, Avenue Pasteur, 6, 1300 Wavre, Belgium; 17 Wallenberg Centre for Molecular and Translational Medicine, University of Gothenburg, 405 30 Gothenburg, Sweden; 18 Institute of Neuroscience and Physiology, Sahlgrenska Academy, University of Gothenburg, 405 30 Gothenburg, Sweden; 19 Dementia Research Centre, Queen Institute of Neurology, University College London, London, WC1N 3BG UK; 20 Laboratory of Neuroimaging of Aging (LANVIE), University of Geneva, 1205 Geneva, Switzerland; 21 Geneva Memory Center, Geneva University Hospitals, 1205 Geneva, Switzerland; 22 German Center for Neurodegenerative Diseases (DZNE), 53127 Bonn, Germany; 23 Division of Clinical Geriatrics, Centre for Alzheimer Research, Department of Neurobiology, Care Sciences, and Society (NVS), Karolinska Institutet, 171 77 Stockholm, Sweden; 24 Theme Inflammation, Karolinska University Hospital, Stockholm, 171 77 Sweden; 25 Theme Aging, Karolinska University Hospital, Stockholm, 171 77 Sweden; 26 Kuopio University Hospital, 70210 Kuopio, Finland; 27 Division of Clinical Geriatrics, Centre for Alzheimer Research, Department of Neurobiology, Care Sciences, and Society (NVS), Karolinska Institutet, 171 77 Stockholm, Sweden; 28 Imperial College London, London, SW7 2AZ UK; 29 University of Edinburgh, Edinburgh, EH8 9YL UK; 30 Barcelonaβeta Brain Research Center (BBRC), Pasqual Maragall Foundation, 08005 Barcelona, Spain; 31 H. Lundbeck A/S, 2500 Copenhagen, Denmark; 32 Janssen Research and Development, Titusville, NJ 08560 USA; 33 Life Molecular Imaging, 13353 Berlin, Germany; 34 GE Healthcare, Pharmaceutical Diagnostics, Amersham, HP7 9LL UK; 35 Institutes of Neurology and Healthcare Engineering, UCL, London, WC1N 3BG UK; 36 Department of Psychiatry and Neuropsychology, School for Mental Health and Neuroscience, Alzheimer Center Limburg, Maastricht University, Maastricht, 6229 ER The Netherlands; 37 Clinical Memory Research Unit, Department of Clinical Sciences, Lund University, 221 00 Malmö, Sweden

**Keywords:** Alzheimer’s disease, Preclinical, Recruitment, Enrollment barriers, Amyloid PET, Clinical trial

## Abstract

**Background:**

The mismatch between the limited availability versus the high demand of participants who are in the pre-dementia phase of Alzheimer’s disease (AD) is a bottleneck for clinical studies in AD. Nevertheless, potential enrollment barriers in the pre-dementia population are relatively under-reported. In a large European longitudinal biomarker study (the AMYPAD-PNHS), we investigated main enrollment barriers in individuals with no or mild symptoms recruited from research and clinical parent cohorts (PCs) of ongoing observational studies.

**Methods:**

Logistic regression was used to predict study refusal based on sex, age, education, global cognition (MMSE), family history of dementia, and number of prior study visits. Study refusal rates and categorized enrollment barriers were compared between PCs using chi-squared tests.

**Results:**

535/1856 (28.8%) of the participants recruited from ongoing studies declined participation in the AMYPAD-PNHS. Only for participants recruited from clinical PCs (*n* = 243), a higher MMSE-score (*β* =  − 0.22, OR = 0.80, *p* < .05), more prior study visits (*β* =  − 0.93, OR = 0.40, *p* < .001), and positive family history of dementia (*β* = 2.08, OR = 8.02, *p* < .01) resulted in lower odds on study refusal. General study burden was the main enrollment barrier (36.1%), followed by amyloid-PET related burden (PC_research_ = 27.4%, PC_clinical_ = 9.0%, *X*^*2*^ = 10.56, *p* = .001), and loss of research interest (PC_clinical_ = 46.3%, PC_research_ = 16.5%, *X*^*2*^ = 32.34, *p* < .001).

**Conclusions:**

The enrollment rate for the AMYPAD-PNHS was relatively high, suggesting an advantage of recruitment via ongoing studies. In this observational cohort, study burden reduction and tailored strategies may potentially improve participant enrollment into trial readiness cohorts such as for phase-3 early anti-amyloid intervention trials.

The AMYPAD-PNHS (EudraCT: 2018–002277-22) was approved by the ethical review board of the VU Medical Center (VUmc) as the Sponsor site and in every affiliated site.

**Supplementary Information:**

The online version contains supplementary material available at 10.1186/s13195-023-01332-4.

## Background

Alzheimer’s disease (AD) is characterized by the presence of amyloid-β plaques and neurofibrillary tangles in the brain, which lead to progressive neurodegeneration, and functional and cognitive impairment [1]. AD is increasingly recognized as a *continuum* in which pathophysiological changes occur many years before the onset of dementia [2, 3]. Individuals in the prodromal phase (presence of pathophysiological changes and mild cognitive impairment [MCI]) and preclinical phase (presence of pathophysiological changes without objective cognitive impairment) are considered an essential population in the ongoing efforts to understand the natural course of AD and develop successful disease-modifying therapies [2, 4–6], such as the recently Food and Drug Administration (FDA)-approved anti-amyloid antibodies aducanumab [7] and lecanemab [8]. A recent meta-analysis on the global prevalence across the AD *continuum* estimates the number of individuals in the prodromal and preclinical phases to be 69 and 315 million, respectively [9]. These numbers emphasize the major public health need posed by AD but also show that the pool of potentially eligible participants for intervention trials is larger than previously assumed. However, as preclinical individuals are by default not (yet) involved in a medical setting, it remains challenging to reach, recruit, and retain them directly into ongoing clinical trials [10, 11]. The resulting mismatch between the number of available versus required participants for clinical trials leads to underpowered study results and hence to scientific, financial, and ethical consequences [10, 12–14]. A proposed strategy to reduce this mismatch, is to build trial readiness cohorts as initiated by among others the Global Alzheimer Platform (GAP) initiative [4, 15]. It is essential to reduce enrollment failure and increase retention rates across potential trial readiness cohorts to optimally use this infrastructure for effective enrollment of preclinical individuals. Previously identified barriers to research participation are various and differ depending on the study design [16, 17] and sociodemographic features of the target population, such as sex, age, education, and clinical status [18–21]. Participation is generally driven by the extent of personal interest (e.g*.*, a drive to advance science or wanting feedback about own health status) and may be hindered by logistical issues (e.g., time-investment and traveling), study burden, distress caused by cognitive testing, one’s perceived health, personal circumstances (e.g., work status, being a caregiver), or a lack of understanding of study information [16, 17, 20, 22–30]. Generally, people seem to be more hesitant to participate in therapeutic clinical trials due to more burdensome study procedures required to measure treatment effects, such as repeated positron emission tomography (PET) imaging [25, 31, 32]. This burden may weigh even heavier in a symptom-free population [25]. However, literature on motivations or barriers to participate in clinical research has focused on populations consisting (largely) of individuals who have symptoms [20, 26, 27, 29, 30], on hypothetical study designs [16, 24, 28, 31], or on a relatively small number of qualitative interviews [23] in preclinical populations.

The Amyloid Imaging to Prevent AD (AMYPAD) Prognostic and Natural History Study (PNHS) is a unique opportunity to study enrollment barriers in the pre-dementia population. The AMYPAD-PNHS was a prospective observational study aiming to investigate the role of amyloid-PET imaging as a predictor of cognitive progression. To this end, the AMYPAD-PNHS included individuals without a dementia diagnosis from the complete AD risk spectrum (i.e., individuals with negative, gray zone, and positive AD biomarkers) and followed their clinical progression over time [33]. Even though the AMYPAD-PNHS itself was not an interventional clinical trial, the enrollment barriers for this observational study may be informative for phase 3 early anti-amyloid intervention trials, which is a timely topic given the recent breakthroughs in anti-amyloid therapies [7, 8]. Firstly, while the AMYPAD-PNHS did not prescribe any pharmacological agents, participation did require the injection of a radioactive tracer, which may give rise to mild worries about invasiveness and side effects. These worries may be even stronger for current phase 3 pharmacological agents [26, 28]. Secondly, participation in the AMYPAD-PNHS involved amyloid-PET imaging, which is a previously reported as a barrier to participate in clinical trials that utilize this imaging technique as an outcome measure of treatment efficacy [25, 28, 31]. Moreover, the AMYPAD-PNHS has recruited participants from 10 parent cohorts (PCs) with characteristically different samples (Supplementary Table S1) which are distributed across seven European countries (Netherlands, Belgium, France, Spain, Switzerland, Sweden, UK). Altogether this has yielded a sample of 1856 eligible subjects recruited from varied and international PCs, of which 1321 consented into the AMYPAD-PNHS and 535 declined. The current study aims to identify the (potentially population-specific) main enrollment barriers for a prospective, multicenter observational amyloid-PET biomarker study. This study specifically included individuals without a dementia diagnosis from existing observational PCs that recruited either cognitively healthy subjects from general society (“research PCs”) or patients with subjective cognitive decline or mild cognitive impairment from a clinical setting (“clinical PCs”). Awareness of these enrollment barriers may potentially aid participant enrollment into trial readiness cohorts such as for phase-3 early anti-amyloid intervention trials.

## Methods

### The AMYPAD-PNHS recruitment strategy and target populations

The AMYPAD-PNHS is a well-phenotyped longitudinal cohort of subjects of  ≥ 50 years of age without a dementia diagnosis. Participants were recruited from PCs that (1) recruited this target population and (2) collected information on domains of AD risk. Eligible subjects were introduced to the AMYPAD-PNHS via their PC and subsequently received the Participation Information Form (PIF) and verbal explanation until all information was deemed understood. Informed consent was obtained on-site ≥ 7 days after receiving the PIF.

Importantly, the AMYPAD-PNHS aimed to include individuals across the complete AD risk spectrum (i.e., individuals with negative, gray zone, and positive AD biomarkers). Furthermore, the PCs differ in composition due to the implementation of local enrollment criteria, and recruitment strategies. However, a general distinction can be made between research cohorts (i.e., those recruiting mainly cognitively healthy subjects from general society) or clinical cohorts (i.e., those recruiting patients with subjective cognitive decline or mild cognitive impairment from a clinical setting).

Study procedures during participation in the PCs were comparable across cohorts and generally included extensive MRI, neuropsychological assessment, and sometimes a lumbar puncture. Participation in the AMYPAD-PNHS always involved one or two visits including an amyloid-PET scan (approximately 2 h), accompanied by MRI (approximately 10 min), and often neuropsychological assessments (approximately 90 min) as part of the PC or as part of the PNHS data collection. For a complete description of the AMYPAD-PNHS recruitment strategy and study procedures, we refer to the AMYPAD-PNHS design paper [33].

The AMYPAD-PNHS (EudraCT: 2018–002277-22) was approved by the ethical review board of the VU Medical Center (VUmc) as the Sponsor site and in every affiliated site. The study was conducted following the Protocol and the Declaration of Helsinki and Good Clinical Practice.

### Parent cohorts

The AMYPAD-PNHS recruited participants from ten PCs: (1) the European Prevention of AD Longitudinal Cohort Study (EPAD-LCS), (2) the European Medical Information Framework for AD 60 +  + /TWINS (EMIF-AD 60 +  + /TWINS), (3) EMIF-AD 90 + , (4) the for Alzheimer and Family study (ALFA +), (5) the Fundació ACE Healthy Brain Initiative (FACEHBI), (6) the Flemish Prevent AD Cohort KU Leuven (F-PACK), (7) the Université Catholique de Louvain (UCL-2010–412 cohort), (8) the Microbiota cohort, (9) the AMYPAD Diagnostic patient management study (DPMS, VUmc only), and (10) H70.

The research cohorts are the EPAD-LCS (*n* = 921), the ALFA + (*n* = 282), F-PACK (*n* = 91), EMIF-AD 60 +  + /TWINS (*n* = 185), and H70 (*n* = 16), while clinical cohorts are the UCL-2010–412 (*n* = 59), EMIF-AD 90 + (*n* = 27), AMYPAD-DPMS Amsterdam (*n* = 47), Microbiota (*n* = 58), and FACEHBI (*n* = 170). The general characteristics of the PCs are summarized from available literature [34–42] in Supplementary Table S1.

### Sociodemographic and clinical characteristics

Sociodemographic and clinical characteristics were obtained from the integrated AMYPAD-PNHS database available through the AD Data Initiative (ADDI) platform and contain data actively collected within the AMYPAD-PNHS and historical data shared by PCs. Historical data from PCs was always shared for participants who consented to AMYPAD-PNHS but, if allowed by the PC, could also be integrated when the participant declined the AMYPAD-PNHS. Variables that change over time were matched to the time of decline or consent, estimated based on the timepoint of registration on the local enrollment logs. If multiple months of recruitment were registered on one log, the median month (maximum deviation ± 11 months) or the timepoint of screening was chosen as an indication.

### Reasons for enrollment failure on the AMYPAD-PNHS enrollment logs

All recruiting sites kept track of screening/enrollment logs, which included whether a subject consented to the AMYPAD-PNHS and reasons for refusing participation. The standardized screening/enrollment log provided six possible reasons to decline participation, namely (1) radiation concerns, (2) claustrophobia, (3) does not want to travel, (4) does not want to be involved in AMYPAD, (5) waiting to go into a proof of concept, and (6) other reasons which could be described by the local investigator. Following the last-patient-in date of the AMYPAD-PNHS (30th of April 2022), all screening/enrollment logs were collected and reviewed by the study sponsor at the VUmc.

**Fig. 1:**
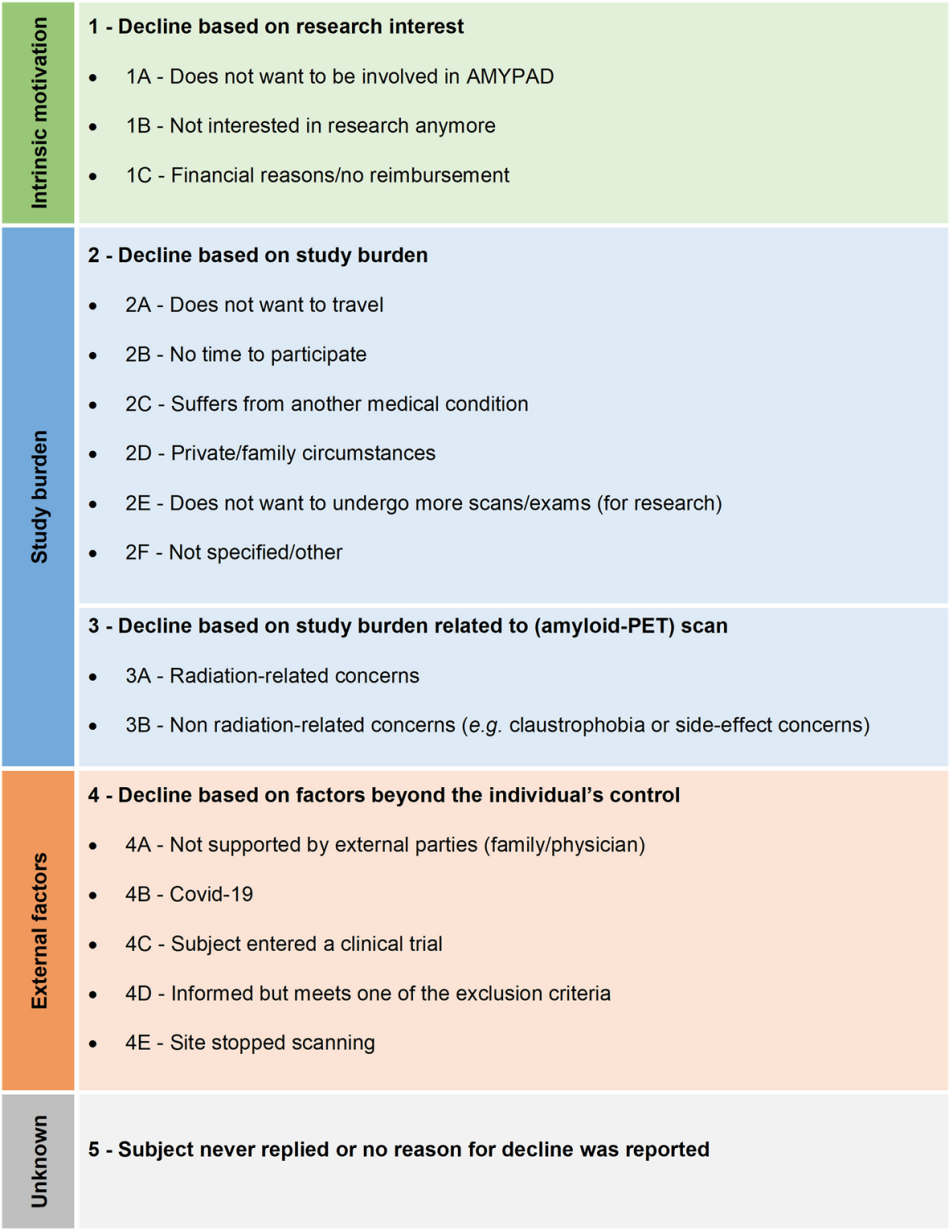
Classification of given reasons to decline participation in the AMYPAD-PNHS

Of the 535 subjects who declined the AMYPAD-PNHS, 348 subjects reported at least one reason in the category “other”. Therefore, reasons to decline participation in the AMYPAD-PNHS were relabeled according to the categories and sub-categories as shown in Fig. 1. Based on a read-through of the data and previous literature [16, 17, 22, 24–28], sixteen subcategories were identified which were grouped under four main categories: (1) research interest, (2) study burden in general, (3) study burden related to (amyloid-PET) scan acquisition, and (4) external factors beyond the individual’s control. Each subject could provide ≥ 1 reason(s) for decline and may therefore be assigned to multiple main categories and/or to multiple sub-categories within a main category. The assignment of each participant to ≥ 1 (sub)categories was conducted by two independent assessors.

### Statistical analyses

Statistical analyses were performed using IBM SPSS Statistics, Version 29.0. Sociodemographic and clinical characteristics were compared between subjects that consented and declined participation across individual cohorts and between clinical and research PCs. Chi-squared tests were used for dichotomous variables (sex, dementia family history), independent sample *T*-tests for continuous variables (age, years of education, MMSE-score), and a non-parametric Mann–Whitney U test for the number of prior visits in PC. Subsequently, logistic regressions were performed to identify the sociodemographic and clinical predictors for enrollment failure from clinical and research PCs separately. Both models included participant status (consent = 0, decline = 1) as the dependent variable and age, sex, years of education, MMSE-score, dementia in family history (none/ ≥ 1 of the parents), and the number of prior visits as independent variables. Since external factors (Fig. 1) were expected to universally affect participants irrespective of socio-demographic factors, participants assigned to this reason of decline were excluded from the analyses (*n* = 95).

Chi-squared tests were performed to compare study refusal rates between PC(-type)s and to determine whether the prevalence of a given reason for decline was significantly different between (1) clinical versus research PCs and (2) individual PCs. The significance threshold was set to* p* < 0.05.

## Results

### Group differences between individuals who consented and declined

**Fig. 2:**
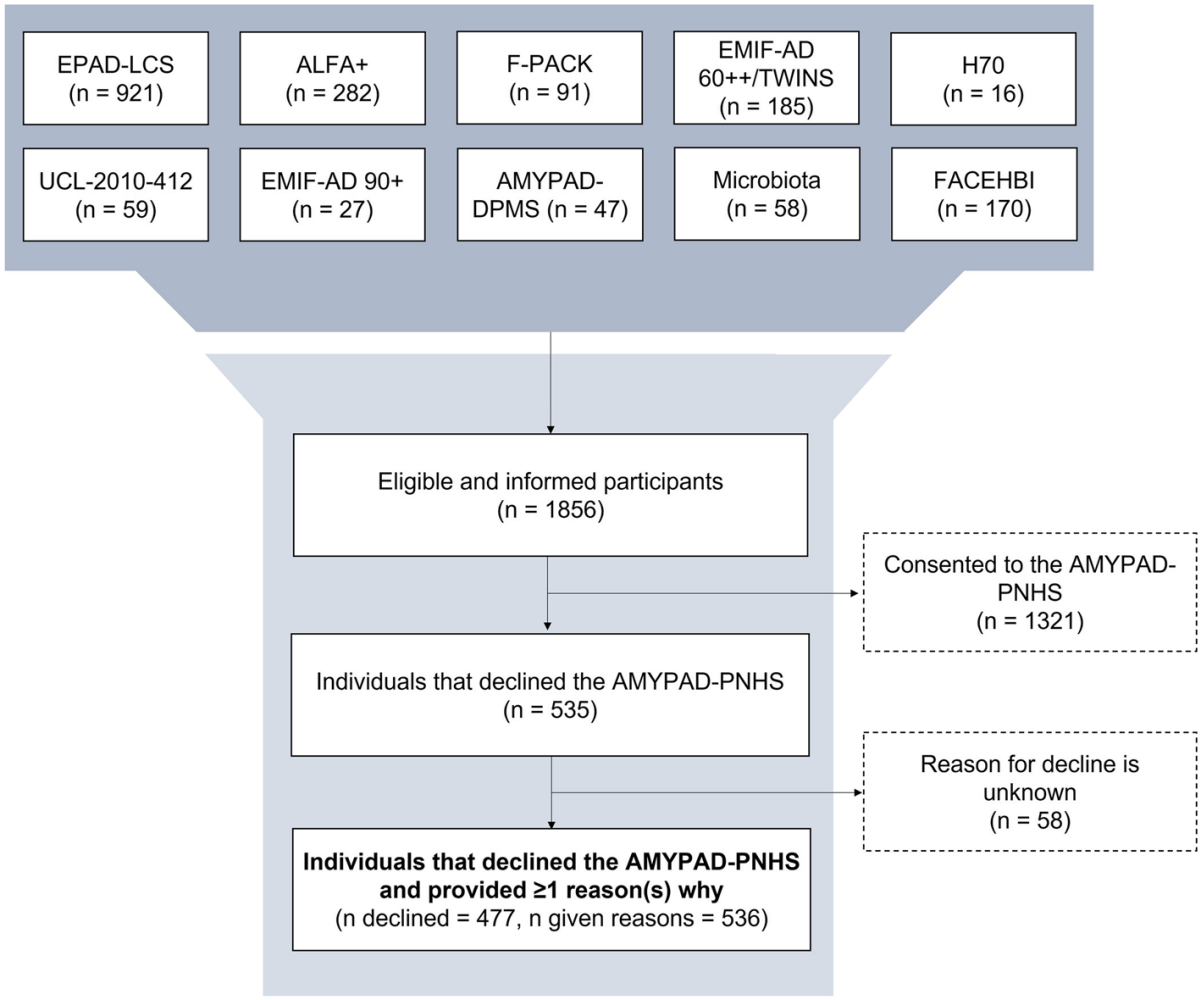
The AMYPAD-PNHS flowchart for recruitment and enrollment

In total, 1856 individuals were informed for participation in the AMYPAD-PNHS, of which 1321 (71.2%) consented and 535 (28.8%) declined (Fig. 2). Socio-demographics are reported in Table 1. Missing data from the integrated AMYPAD-PNHS database is reported in Supplementary Table S2. Overall, individuals who declined the study were significantly older, less educated, and scored significantly lower on the MMSE. This was fully driven by the clinical PCs, where individuals who declined the study less often reported a positive family history of dementia and completed fewer prior visits in their PC (Table 1).

**Table 1:** Sociodemographic and clinical differences between individuals who consented and declined participation in the AMYPAD-PNHS

	**Research cohorts**			**Clinical cohorts**			**All cohorts**		
	**Consented**	**Declined**	**Test statistic**	**Consented**	**Declined**	**Test statistic**	**Consented**	**Declined**	**Test statistic**
**Female**	572/1001 (57.1)	173/287 (60.3)	ns	156/283 (55.1)	22/54 (40.7)	ns	728/1284 (56.7)	195/341 (57.2)	ns
**Family history (≥ 1 parent dementia)**	662/976 (67.8)	190/287 (66.2)	ns	118/216 (54.6)	4/31 (12.9)	*X*^2^(1) = 18.88, *p* < .001	780/1192 (65.4)	194/318 (61.0)	ns
**Age (years)**	67.6 ± 7.33	68.3 ± 8.00	ns	72.0 ± 9.96	77.1 ± 8.46	*t*(335) = -3.53, *p* < .001	68.6 ± 8.18	69.7 ± 8.67	*t*(1623) = -2.18, *p* < .05
**Education (years)**	14.6 ± 3.78	14.3 ± 3.89	ns	15.0 ± 4.30	13.3 ± 4.07	*t*(335) = 2.75, *p* < .01	14.7 ± 3.90	14.2 ± 3.93	*t*(1623) = 2.36, *p* < .05
**MMSE (score)**	28.1 ± 1.54	28.6 ± 1.48	ns	28.4 ± 1.98	27.0 ± 2.44	*t*(335) = 3.75, *p* < .001†	28.7 ± 1.65	28.4 ± 1.76	*t*(1622) = 3.13, *p* < .01†
**Mean nr. of prior visits**^**‡**^	3 ± 1	3 ± 1	ns	6 ± 2	4 ± 2	*U* = 4132.5, *p* < .001	3 ± 1	3 ± 2	ns

Continuous variables are reported in mean ± sd and categorical variables in *n* (%). As external factors (Fig. 1) are expected to universally affect participants irrespective of individual characteristics, participants assigned to this reason of decline were excluded from these analyses (*n* = 95). Not all PCs provided data for both consented and declined participants: missing cases are reported in Supplementary Table S2. ^†^Equal variances not assumed. ^‡^Reported in median ± IQR and Mann–Whitney *U* test due to non-normality of the variable. *MMSE*, Mini-Mental State Examination

To identify the sociodemographic and clinical predictors for study refusal, logistic regressions were performed for clinical and research PCs separately (Table 2). The Hosmer–Lemeshow goodness-of-fit was non-significant for both the research model (*X*^2^(8) = 7.02, ns) and clinical model (*X*^2^(8) = 7.02, ns) indicating adequate model fits. For research PCs, the model explained 9% (Nagelkerke *R*^2^) of the variance in participant status and correctly classified the enrollment status in 77.3% of the cases. However, none of the predictors in the research PC model were significant contributors. For clinical PCs, the model explained 44% (Nagelkerke *R*^2^) of the variance in participant status and correctly classified the enrollment status in 89.8% of the cases. A higher MMSE score and a higher number of prior study visits in the PC significantly lowered the odds of declining participation in the AMYPAD-PNHS (*β* =  − 0.22, *p* < 0.05, OR = 0.80 [95%-CI = 0.64–1.00], and *β* =  − 0.93, *p* < 0.001, OR = 0.40 [95%-CI = 0.27–0.58], respectively), while the absence of a family history of dementia significantly increased the odds on declining participation the AMYPAD-PNHS (*β* = 2.08, *p* < 0.01, OR = 8.02 [95%-CI = 2.03–31.8]).

**Table 2:** Binary logistic regression model including sociodemographic and clinical predictors for refusing participation in the AMYPAD-PNHS

	**Research cohorts**					**Clinical cohorts**				
	**B**	**SE(B)**	**Wald**	***p*-value**	**Exp(B) [95%-CI]**	***B***	**SE(B)**	**Wald**	***p*-value**	**Exp(B) [95%-CI]**
**Age (years)**	0.01	0.01	2.05	0.15	1.01 [0.92–1.18]	− 0.01	0.02	0.16	0.69	0.99 [0.95–1.04]
**Sex (female = 1)**	0.14	0.14	1.00	0.32	1.15 [0.87–1.52]	− 0.44	0.51	0.72	0.39	0.65 [0.24–1.76]
**Education (years)**	-0.01	0.02	0.68	0.41	0.99 [0.95–1.02]	− 0.07	0.06	1.24	0.27	0.94 [0.83–1.05]
**MMSE (score)**	-0.06	0.04	1.99	0.16	0.94 [0.86–1.02]	− 0.22	0.11	3.89	0.048*	0.80 [0.64–1.00]
**Family history (none of the parents = 1)**	-0.01	0.15	0.01	0.93	0.99 [0.73–1.33]	2.08	0.70	8.79	0.003*	8.02 [2.03–31.8]
**Number of prior visits**	0.04	0.07	0.39	0.53	1.04 [0.92–1.18]	− 0.93	0.19	23.3	0.000*	0.40 [0.27–0.58]

Logistic models included participant status (consent = 0, decline = 1) as dependent variable and age, sex, years of education, MMSE score, dementia in family history (none or ≥ 1 of the parents), and the number of prior visits as independent variables. As external factors (Fig. 1) are expected to universally affect participants irrespective of individual characteristics, participants assigned to this reason of decline were excluded from these analyses (*n* = 95). Research cohorts: *n* = 1263 (*n* consent = 976, *n* decline = 287). Clinical cohorts: *n* = 243 (*n* consent = 215, *n* decline = 31). Asterisks indicate predictors that add significantly to the prediction (*p* < .05)

### Enrollment (barriers) within the AMYPAD-PNHS

**Fig. 3:**
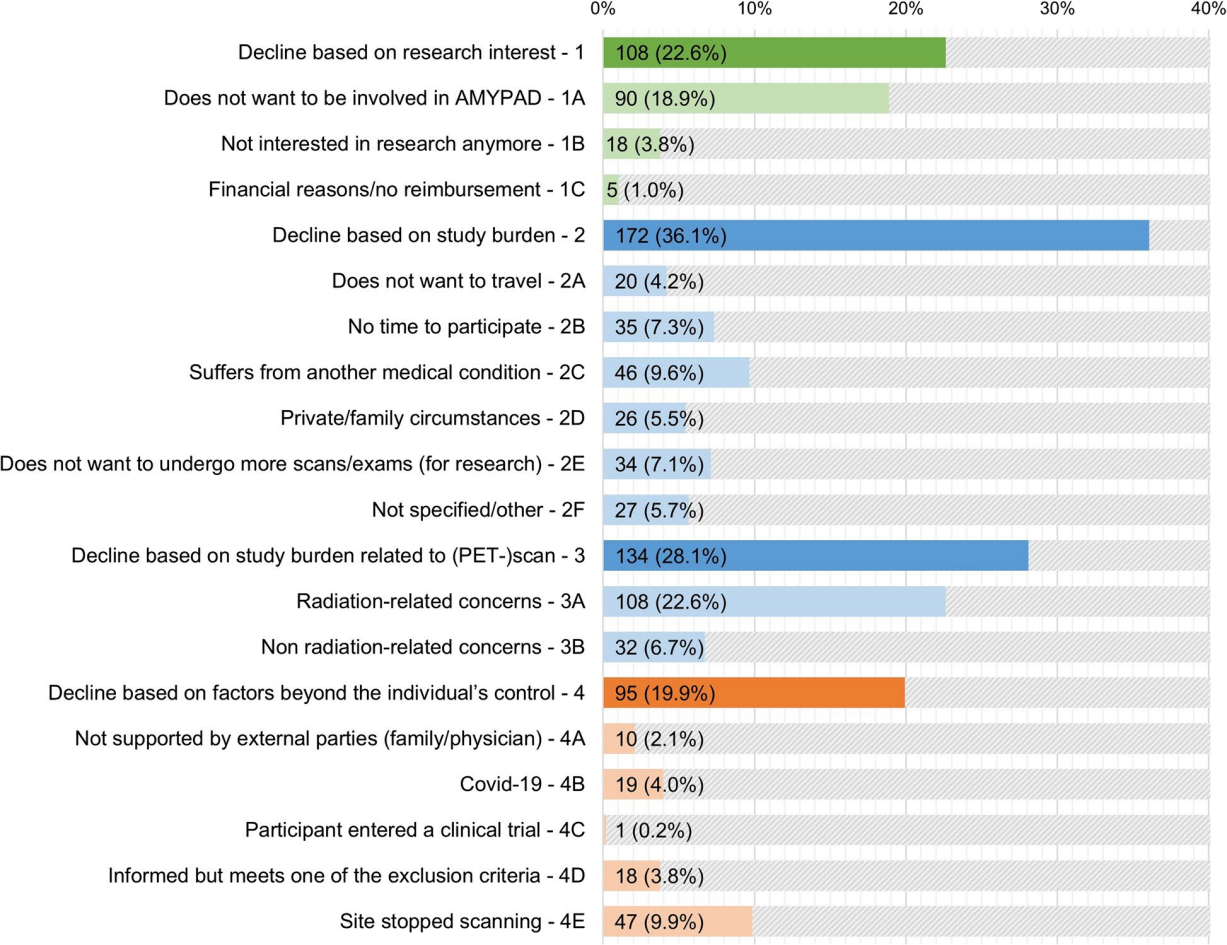
Absolute and relative frequency of reported reasons not to enroll in the AMYPAD-PNHS. Colored bars: the absolute number of times a reason is reported (% relative to the total number of subjects, *n* = 477). Gray bars: the remaining subjects that did not report this reason, truncated at 50% for visualization purposes (100% = the total number of 477 subjects). Each subject could provide ≥ 1 reason(s) supporting their refusal and may therefore be assigned to multiple main categories and/or to multiple sub-categories within a main category. A total of 509 main categories and 536 sub-categories have been reported

Four hundred seventy-seven of 535 (89.2%) of the individuals who declined participation in the AMYPAD-PNHS provided ≥ 1 reason(s), leading to a total of 536 reasons not to enroll. The absolute and relative prevalence of each main category and corresponding subcategories is shown in Fig. 3. Most of the given reasons to decline were related to the general study burden (*n* = 172, 36.1%), followed by the study burden related to the amyloid-PET scan (*n* = 134, 28.1%), research interest (*n* = 108, 22.6%), and factors beyond the individual’s control (*n* = 95, 19.9%). Radiation-related concerns (*n* = 108, 22.6%) and not wanting to be involved in AMYPAD (*n* = 90, 18.9%) were the most prevalent subcategories among individuals who declined the AMYPAD-PNHS.

### Enrollment (barriers) across PCs

**Fig. 4:**
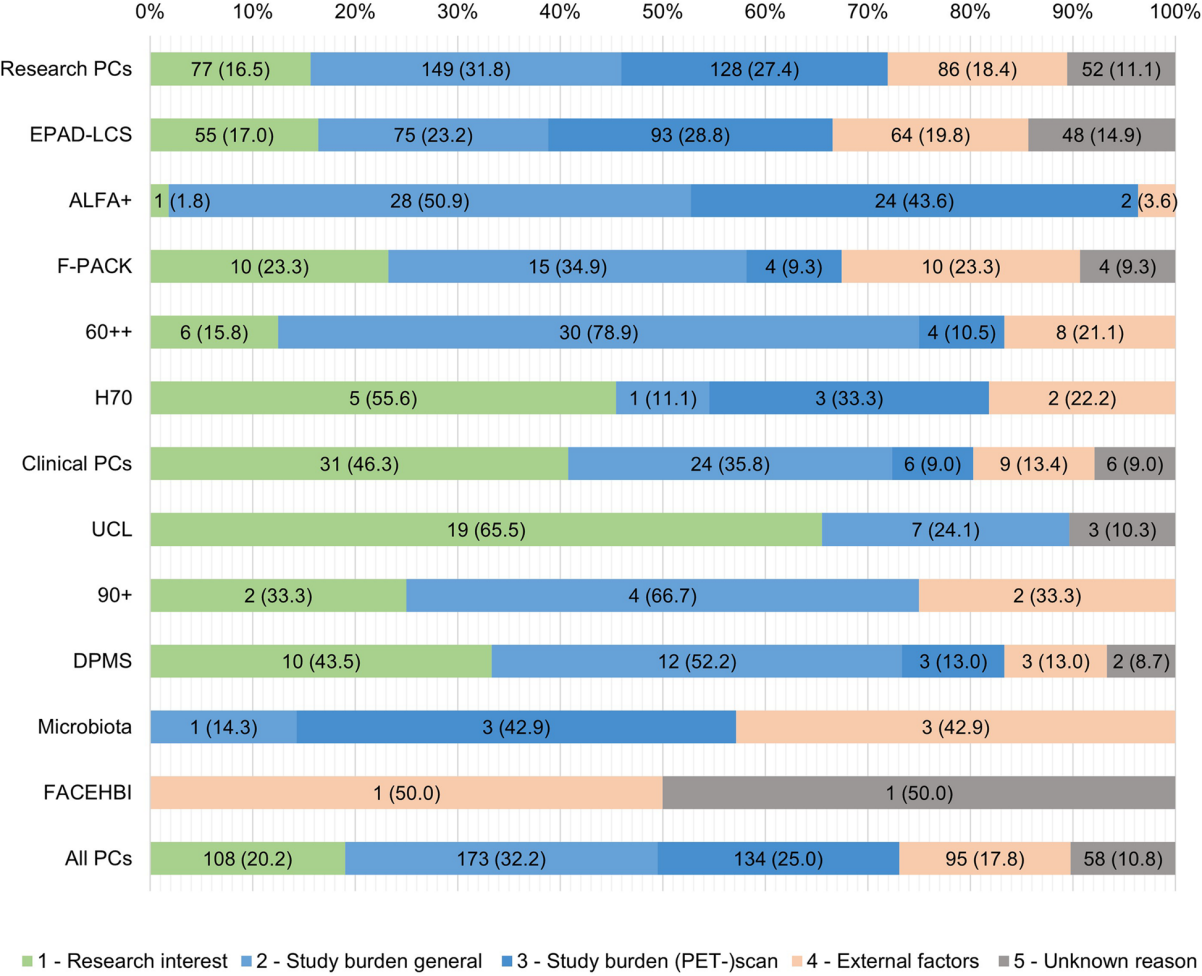
Absolute and relative frequency of reasons not to enroll in the AMYPAD-PNHS for all PCs. Research PCs are EPAD-LCS, ALFA + , F-PACK, EMIF-AD 60 +  + ; Clinical PCs are UCL-2010–412, EMIF-AD 90 + , AMYPAD DPMS, Microbiota, and FACEHBI. Abbreviations: 60 +  +  = EMIF-AD 60 +  + /TWINS; UCL = UCL-2010–412; DPMS = AMYPAD DPMS; 90 +  = EMIF-AD 90 +

The refusal rate across research PCs (468/1495 (31.3%)) was significantly higher than across clinical PCs (67/361 (18.6%); *X*^*2*^(1) = 23.02, *p* < 0.001) (Table 3). This difference remained statistically significant when declines due to external factors were excluded from the analysis (*X*^*2*^(1) = 14.86, *p* < 0.001) (Supplementary Table S3). A loss of research interest was relatively more prevalent in clinical PCs compared to research PCs (*X*^*2*^(1) = 32.34, *p* < 0.001), while study burden related to the amyloid-PET scan was relatively less prevalent (*X*^*2*^(1) = 10.56, *p* = 0.001) (Table 3 and Fig. 4).

**Table 3:**
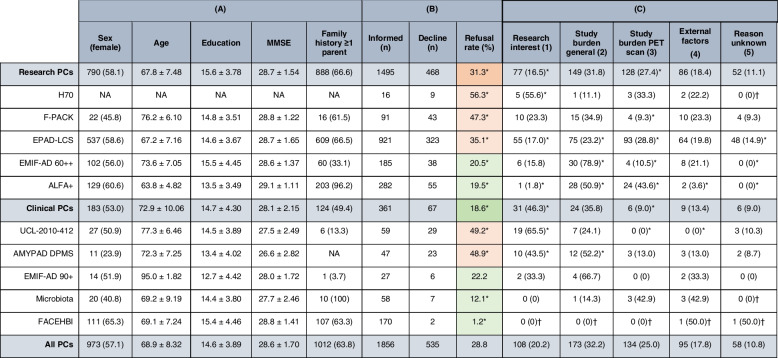
Demographics, study refusal rates, and main reasons for decline for all parent cohorts

Comparisons for refusal rates (df = 1, *n* = 1856) and the prevalence of a given reason for refusal (df = 1, *n* = 535) are performed for a single PC versus all other PCs (white rows) and research versus clinical PCs (gray rows). (A) Continuous variables are reported in mean ± sd and categorical variables in n (%). Not all PCs provided data for both consented and declined participants: missing cases are reported in supplementary Table S2. (B) Refusal rate (%) = *n* informed / *n* declined for research PCs (EPAD-LCS, ALFA + , F-PACK, EMIF-AD 60 + +) and clinical PCs (UCL-2010–412, EMIF-AD 90 + , AMYPAD DPMS, Microbiota, FACEHBI). Orange indicates an above-average refusal rate and green a below-average refusal rate. Asterisks indicate a significant difference (*p* < .05) between the refusal rate in research versus clinical PCs (darker shade) or for this main PC versus all other PCs (lighter shade) according to a chi-squared test (df = 1, *n* = 1856). (C) The *n* (%) is reported which shows the absolute and relative number of times a subcategory for decline was grouped under one of the main categories 1–5 described in Figs. 1 and 4. Asterisks indicate significant differences (*p* < .05) between the prevalence of a given reason between research versus clinical PCs (gray) or for this main PC versus all other PCs (white) according to a chi-squared test (df = 1, *n* = 535). Crosses indicate unreliable chi-squared testing based on violation of the assumptions (≥ 80% of the cells had an expected value ≤ 5 or one cell had an expected value of < 1) [53]. *PC*, parent cohort; *PET*, positron emission tomography; *MMSE*, Mini-Mental State Examination; *NA*, not applicable

The study refusal rates across the separate PCs appeared highly variable. Refusal rates were relatively high in H70 (9/16 (56.3%); *X*^*2*^(1) = 5.91, *p* < 0.05), UCL-2010–412 (29/59 (49.2%); *X*^*2*^(1) = 12.27, *p* < 0.001), AMYPAD-DPMS (23/47 (48.9%); *X*^*2*^(1) = 9.51, *p* < 0.01), F-PACK (43/91 (47.3%); *X*^*2*^(1) = 15.84, *p* < 0.001), and EPAD-LCS (323/921 (35.1%); *X*^*2*^(1) = 34.75, *p* < 0.001). In these PCs with a high refusal rate, a loss of research interest was significantly more often reported in H70 (*X*^*2*^(1) = 7.11, *p* < 0.01), UCL-2010–412 (*X*^*2*^(1) = 39.10, *p* < 0.001), and AMYPAD DPMS (*X*^*2*^(1) = 8.09, *p* < 0.01). For AMYPAD-DPMS, this was accompanied by a relatively high report of decline due to the general study burden (*X*^*2*^(1) = 4.32, *p* < 0.05). In contrast, a loss of research interest was less prevalent in EPAD-LCS (*X*^*2*^(1) = 5.05, *p* < 0.05) and the high study refusal rate was rather due study burden related to the amyloid-PET scan (*X*^*2*^(1) = 6.09, *p* < 0.05) (Table 3 and Fig. 4).

Refusal rates were relatively low in EMIF-AD 60 +  + (38/185 (20.5%); *X*^*2*^(1) = 6.87, *p* < 0.01), ALFA + (55/282 (19.5%); *X*^*2*^(1) = 14.08, *p* < 0.001), Microbiota (7/58 (12.1%); *X*^*2*^(1) = 8.19, *p* < 0.01), and FACEHBI (2/170 (1.2%); *X*^*2*^(1) = 69.73, *p* < 0.001). The total number of declines for Microbiota and FACEHBI were too small for statistical comparisons. For the remaining PCs with a low refusal rate, the general study burden was relatively often reported in EMIF-AD 60 +  + (*X*^*2*^(1) = 40.62, *p* < 0.001) and ALFA + (*X*^*2*^(1) = 9.66, *p* < 0.01). In the ALFA + cohort, there was little loss of research interest (*X*^*2*^(1) = 12.84, *p* < 0.001), but the general study burden (*X*^*2*^(1) = 9.66, *p* < 0.01) and study burden related to the (amyloid-PET) scan (*X*^*2*^(1) = 11.28, *p* = 0.001) appeared to be more prevalent. Similarly, the general study burden was a significant contributor to the decline in the EMIF-AD 60 +  + cohort (*X*^*2*^(1) = 40.62, *p* < 0.001), but this was less often related to the (amyloid-PET) scan (*X*^*2*^(1) = 4.59, *p* < 0.05) (Table 3 and Fig. 4).

The study refusal rate and reported reasons of the EMIF-AD 90 + cohort were not significantly different (Table 3 and Fig. 4).

## Discussion

This study identified the main enrollment barriers for a prospective, multicenter, observational amyloid-PET biomarker study including individuals without a dementia diagnosis. The enrollment rate in the AMYPAD-PNHS was high (71.2%), suggesting that recruitment from a platform of ongoing studies provides an advantage over population-based recruitment, which has been previously illustrated by a sharp increase in the number needed to prescreen in population-based cohorts versus clinical and research in-person cohorts [43]. In this population recruited from ongoing studies, enrollment from research PCs appears largely independent of group characteristics, whereas recruitment from clinical PCs is more successful when subjects have less cognitive impairment, completed more prior study visits in their PC, and had a positive family history of dementia. Of note, MMSE scores within the normal range are intrinsic to a sample of cognitively healthy volunteers, and these individuals are generally younger and more highly educated. As a result, more extreme values of the reported variables with predictive value for refusal are likely underrepresented in research PCs. Based on the classification of reasons for decline (Fig. 1), the perceived general study burden was the main cause for study refusal irrespective of PC type. In addition, the overall study refusal rate in research PCs was high and mostly based on amyloid-PET-related concerns whereas the overall study refusal rate in clinical PCs was lower and mostly due to a loss of research interest. Based on these results, both general and population-specific burden-to-benefit factors affect decision-making to enroll in clinical research.

According to the current results and previous literature in individuals with dementia [20, 26, 27, 29, 30] and without dementia [16, 31], strategies to reduce the general study burden could promote enrollment irrespective of the target population [20]. The AMYPAD-PNHS aimed to limit the general study burden by recruiting participants from PCs to build upon existing data. As a result, the study burden within the AMYPAD-PNHS is lower than for current clinical trials which often require repeated visits and repeated drug infusions (e.g., for lecanemab [8]). Based on this difference and the significant impact observed for study burden, the refusal rate for the AMYPAD-PNHS is most likely an underestimation of the refusal rate for these clinical trials. Nevertheless, the additional burden of an amyloid-PET scan (and MRI and neuropsychological assessment for some PCs) explained a large portion of declines. Potentially, participants already reached the limit of the study burden they were willing/able to handle, which could be resolved by improved collaborations to prioritize and combine the most relevant study activities. In addition, participants value being accommodated in logistic aspects (e.g., transport and more flexibility in appointment making) [20, 44, 45] or implementation of online assessment approaches [46].

Aside from a general reduction in study burden, tailored enrollment strategies for research versus clinical populations are relevant to effectively promote enrollment. According to previous literature, individuals who are symptomatic are more likely to enroll in higher-burden studies based on individual expected clinical benefits [17, 24, 31]. The absence of potential individual benefit in cases without symptoms reduces their willingness to enroll in higher-burden study scenarios [31]. Interestingly, in the AMYPAD-PNHS, we also observed relatively low enrollment rates for healthy volunteers compared to clinical subjects. Given the observational nature of the study, this finding cannot be explained by any expected therapeutic benefit. However, these findings and previous literature suggest a general difference in willingness to undergo more burdensome procedures when individuals are asymptomatic. One approach to tip the balance of the burden-to-benefit ratio is to reduce the perceived burden of study participation. For example, enrollment of healthy volunteers could be enhanced by reducing perceived risks through information on the (low) risks of an amyloid-PET scan. Despite the demonstrated safety and benefits of nuclear medicine, the general public’s perception of ionizing radiation remains negatively influenced by historical and socio-psychological factors [47, 48]. Within the AMYPAD-PNHS, subjects were informed about the amyloid-PET scan in the PIF and followed up telephonically. Nevertheless, 27.4% of the declines within research PCs were related to the amyloid-PET scan. Hence, in addition to recent efforts to establish best clinical practices for amyloid-PET disclosure [49, 50], future clinical PET studies should increase their efforts to implement more elaborate strategies to improve understanding of risks and to build trust prior to the PET scan [51].

Whereas enrollment from research PCs was mostly hindered by perceived risks related to the amyloid-PET scan, enrollment from clinical PCs was mostly affected by a loss of research interest. Based on this, implementation of patient engagement strategies could promote enrollment of clinical subjects, especially when they have more cognitive impairment, are less involved in previous research, and have no family history of dementia. Engagement strategies can appeal to known motivations based on (1) personal benefit or (2) altruistic reasons. First, individuals with cognitive impairment can expect a therapeutic benefit from medication in clinical trials [23, 24]. As this is not the case in observational research like the AMYPAD-PNHS, alternative strategies are providing updates on general study progress and enabling access to care and support [18, 23, 52]. Secondly, known altruistic motivations for enrollment include helping a loved one or advancing science for future patients [24]. For example, knowing someone who is living with dementia is a known motivator for clinical trial participation [23] and was a strong predictor for the odds of enrollment. Especially when subjects do not have a family history of dementia, it is essential to convey the message that participation aids future patients and to make participants feel valued by providing tokens of appreciation [17, 18, 23, 24, 31].

Additional lessons regarding engagement strategies can be learned from individual PCs. Study refusal rates across PCs were highly variable and ranged from 1.2% (FACEHBI) to 56.3% (H70) (Table 2). A loss of interest was highly prevalent in all PCs with a higher refusal rate (except EPAD-LCS) and almost absent in the PCs with a lower refusal rate, including the (clinical) FACEHBI cohort. Participants from FACEHBI had already undergone two amyloid-PET scans and MRIs in previous study visits and agreed to undergo a third scan as part of the AMYPAD-PNHS. FACEHBI recruited participants from both the Memory Clinic and the Open House Initiative [53] at Ace Alzheimer Center Barcelona. Ace actively focuses on patient engagement, recruitment, and retention [42, 52] and was the leader of the *Models of patient engagement for Alzheimer’s disease* (MOPEAD) innovative medicines initiatives (IMI) project [54]. This project focused on communication and trust-building with patients and caregivers and on providing perspective after participation [52, 55]. Although these strategies are not necessarily generalizable to all centers, previous literature has shown a positive correlation between the number of implemented engagement categories and retention rates [18, 56], which supports a general effect of engagement strategies and suggests that active efforts to make patients feel valued and supported may prove fruitful in clinical trial enrollment.

Notably, a percentage of loss of enrollment could not be explained by individual motivations or experience of study burden but rather by external factors. A minor group of participants might have been willing to participate, but this decision was not supported by their family or physician or they did not meet the eligibility criteria. A somewhat surprising finding in this category is the seemingly small impact of COVID-19. However, the true impact of COVID-19 is partly reflected by the “site stopped scanning” category because sites could not recruit the backlog of participants that emerged after site closure due to COVID-19 measures or by inability to complete the study procedures *after* enrollment. Importantly, results of PCs with a high report of enrollment failure due to external factors should be interpreted with some caution as these external factors could have caused an under-report of potential individual reasons.

Strengths of the current study lie in the large and varied sample of mostly preclinical subjects and the real-life (rather than hypothetical [16, 24, 28, 31]) enrollment in a study involving study activities that are informative for clinical trial enrollment, such as injection of a radioactive tracer and amyloid-PET imaging. Furthermore, due to the strategy of the AMYPAD-PNHS to recruit only from PCs, all individuals were familiar with scientific research, which makes the sample representative of a so-called trial-ready population [4]. Finally, reasons to decline were relabeled to prevent loss of information and grouped into main categories to improve (statistical) interpretability of the results. Nevertheless, the results of this study should be interpreted in light of some considerations. Firstly, although the AMYPAD-PNHS is, to some extent, comparable to a therapeutic clinical trial, no intervention was provided and enrollment may therefore encompass different considerations. For example, a more cognitively impaired subject from a clinical PC may be less likely to enroll in the AMYPAD-PNHS but could be motivated by potential therapeutic benefits in a clinical trial [23, 24]. Secondly, the current study sample is based on initial consent and potential barriers up until the completion of the study are missing. Thirdly, participants were free to mention multiple decline reasons but were not routinely provided with predefined categories. As a result, the first-mentioned (and likely the main) reasons to decline were reported but this does not definitively exclude the involvement of other considerations. Finally, not all sociodemographic variables were consistently available across all PCs. Constraints in sharing historical data led to missing data, particularly for the group that declined participation in the AMYPAD-PNHS. Furthermore, due to differences in data collection, we could not address previously reported recruitment disparities by race and ethnicity [57, 58] in this study population.

## Conclusions

Based on the current results, recruitment from a platform of ongoing studies provides an advantage over population-based recruitment. When participants are recruited from ongoing studies, their decision to enroll in subsequent clinical research is based on both general (study burden) and population-specific burden-to-benefit factors (amyloid-PET-related concerns in research PCs versus a loss of research interest in clinical PCs). This indicates potential for general enrollment strategies (reducing study burden) and tailored strategies for individuals without symptoms (improved communication around amyloid-PET) or individuals with symptoms (implementation of patient engagement strategies). Across individuals with symptoms, particular focus should be on subjects who have more cognitive impairment, were less involved in previous research, and have no family history. Future studies are required to compare the effectiveness of specific general and tailored strategies in different target populations for clinical trial readiness cohorts [18, 20].

### Supplementary Information

**Additional file 1:** **Table S1.** Literature-based characteristics of the ten parent cohorts within the AMYPAD-PNHS. **Table S2.** Missing data on the AD Data Initiative (ADDI) platform for demographic and clinical characteristics among the individuals that consented and declined participation in the AMYPAD-PNHS. **Table S3.** Study refusal rates and main reasons for decline for all parent cohorts after excluding declines due to external factors.

## Data Availability

Data is available based on reasonable request.

## Abbreviations

AD: Alzheimer’s disease
ADDI: AD Data Initiative
ALFA+: For Alzheimer and Family study
AMYPAD: Amyloid Imaging to Prevent AD
CI: Confidence interval
DPMS: Diagnostic patient management study
EMIF-AD 60 + +: European Medical Information Framework for AD 60 + + /
EPAD-LCS: European Prevention of AD Longitudinal Cohort Study
FACEHBI: The Fundació ACE Healthy Brain Initiative
F-PACK: Flemish Prevent AD Cohort KU Leuven
IMI: Innovative medicines initiatives
MMSE: Mini-Mental State Examination
MOPEAD: Models of patient engagement for Alzheimer’s disease
MRI: Magnetic resonance imaging
Ns: Non-significant
OR: Odds ratio
PC: Parent cohort
PET: Positron emission tomography
PIF: Partipant Information Form
PNHS: Prognostic and Natural History Study
UCL-2010–412 cohort: Université Catholique de Louvain cohort
VUmc: Vrije Universiteit medisch centrum
