# Supplementary material for: Recruitment of pre-dementia participants: main enrollment barriers in a longitudinal amyloid-PET study
Source: Alzheimers Res Ther. 2023 Nov 2;15:189. doi: 10.1186/s13195-023-01332-4 (PMC10621165; doi:10.1186/s13195-023-01332-4)
Supplement: Supplementary file 1 — Additional file 1: Table S1. Literature-based characteristics of the ten parent cohorts within the AMYPAD-PNHS. Table S2. Missing data on the AD Data Initiative (ADDI) platform for demographic and clinical characteristics among the individuals that consented and declined participation in the AMYPAD-PNHS. Table S3. Study refusal rates and main reasons for decline for all parent cohorts after excluding declines due to external factors. [file 13195_2023_1332_MOESM1_ESM.docx]

# Supplementary material

|  | **Research cohorts** | | | | | **Clinical cohorts** | | | | | |
| --- | --- | --- | --- | --- | --- | --- | --- | --- | --- | --- | --- |
|  | **EPAD-LCS^1^** | **ALFA+^2^** | **F-PACK^3^** | **EMIF-AD 60++ ^4^** | **H70^5^** | **UCL-2010-412^6^** | **EMIF-AD 90+^7^** | **AMYPAD DPMS ^8^** | | **Microbiota** | **FACEHBI^9^** |
| **Country** | European^†^ | Spain | Belgium | Netherlands | Sweden | Belgium | Netherlands | Netherlands | | Switzerland | Spain |
| **Target population** | CN | CN | CN | CN | CN | CN/SCD/MCI | CN/aMCI/pAD | SCD | MCI | CN/MCI | SCD |
| **Sample size (n)** | 1065 | 205 | 180 | 204 | 1203 | 160 | 122 | 38 | 50 | NA | 200 |
| **Sex (female)** | 602 (59.0) | 134 (65.4) | 81 (45.0) | 119 (58.0) | 644 (53.5) | 81 (51.0) | 70 (57.4) | 8 (21.0) | 15 (30.0) | NA | 125 (62.5) |
| **Age (years)** | 65.1 ± 7.0 | 61.0 ± 4.7 | 68.6 ± 6.4 | 70.8 ± 7.8 | 70.5 ± NA | 71.4 ± 7.5 | 92.4 ± 2.8 | 66 ± 9^‡^ | 72 ± 10^‡^ | NA | 65.9 ± 7.2 |
| **Education (years)** | 14.5 ± 3.7 | 13.5 ± 3.6 | 14.2 ± 3.4 | 14.9 ± 4.5 | NA | 14.4 ± 4.6 | 11.4 ± 3.4 | 13 ± 6^‡^ | 10 ± 5^‡^ | NA | 14.8 ± 4.7 |
| **MMSE (score)** | 28.7 ± 1.6 | NA | 29.1 ± 0.9 | 28.9 ± 1.2 | 28.8 ± NA | 27.3 ± 1.8 | 27.1 ± 3.1 | 28 ± 2^‡^ | 26 ± 2^‡^ | NA | 29.2 ± NA |

**Table S1 - Literature-based characteristics of the ten parent cohorts within the AMYPAD-PNHS**. Continuous variables are reported in mean ± sd and categorical variables in n (%). †EPAD-LCS is located in Scotland, France, Spain, Netherlands, Belgium, Sweden, Switzerland. ‡Variable reported in median ± IQR. Abbreviations: CN=cognitively normal; SCD=subjective cognitive complaints; aMCI=amnestic mild cognitive impairment; pAD=probable/possible AD, MMSE=mini mental state examination, NA=no published data available. References: 1: 33; 2: 34; 3: 35; 4: 36; 5: 37; 6: 38; 7: 39; 8: 40; 9: 41.

| **Parent cohort** | **Missing cases among consents** | **Missing cases among declines** |
| --- | --- | --- |
| **EPAD-LCS** | 3 (0.5) | 1 (0.4) |
| **ALFA+** | 14 (6.2) | 53 (100) |
| **FACEHBI** | 0 | 0 |
| **EMIF-AD 60++/TWINS** | 2 (1.4) | 1 (3.3) |
| **EMIF AD 90+** | 0 | 0 |
| **AMYPAD-DPMS** | 0 | 0 |
| **F-PACK** | 0 | 33 (100) |
| **UCL-2010-412** | 6 (20) | 0 |
| **Microbiota** | 5 (9.8) | 4 (100) |
| **H70** | 7 (100) | 7 (100) |
| **All PCs** | 37 (2.8) | 99 (22.5) |

**Table S2 – Missing data on the AD Data Initiative (ADDI) platform for demographic and clinical characteristics among the individuals that consented and declined participation in the AMYPAD-PNHS**. Missing cases are reported as n (%) among individuals who consented and declined. As external factors are expected to universally affect participants irrespective of individual characteristics, participants assigned to this reason of decline were excluded from demographic comparisons (n=95) and here. PC = Parent cohort.

|  | **Informed (n)** | **Decline (n)** | **Refusal rate (%)** |
| --- | --- | --- | --- |
| **Research PCs** | 1409 | 382 | 27.1* |
| **H70** | 14 | 7 | 50.0* |
| **F-PACK** | 81 | 33 | 40.7* |
| **EPAD-LCS** | 857 | 259 | 30.2* |
| **EMIF-AD 60++** | 177 | 30 | 16.9* |
| **ALFA+** | 280 | 53 | 18.9* |
| **Clinical PCs** | 352 | 58 | 16.5* |
| **UCL-2010-412** | 59 | 29 | 49.2* |
| **AMYPAD DPMS** | 44 | 20 | 45.5* |
| **EMIF-AD 90+** | 25 | 4 | 16.0 |
| **Microbiota** | 555 | 4 | 7.3* |
| **FACEHBI** | 169 | 1 | 0.60* |
| **All PCs** | 1761 | 440 | 25.0 |

**Table S3 – Study refusal rates and main reasons for decline for all parent cohorts after excluding declines due to external factors.** (A) Refusal rate (%)=n informed / n declined for research PCs (EPAD-LCS, ALFA+, F-PACK, EMIF-AD 60++) and clinical PCs (UCL-2010-412, EMIF-AD 90+, AMYPAD DPMS, Microbiota, FACEHBI). Asterisks indicate significant differences (*p*<.05) between the refusal rate in research versus clinical PCs (grey) or for this main PC versus all other PCs (white) according to a chi-squared test (df=1, n=1764). PC = Parent cohort.
